# The higher certificate in disability practice strengthening rehabilitation in community-oriented primary care

**DOI:** 10.4102/ajod.v14i0.1515

**Published:** 2025-07-31

**Authors:** Shireen Damonse, Nafisa Mayat, Sumaya Gabriels, Eve M. Duncan

**Affiliations:** 1 Department of Health and Rehabilitation Sciences, Faculty of Health Sciences, University of Cape Town, Cape Town, South Africa

## Introduction

Current public health system reforms emphasise community-oriented primary care (COPC) as part of universal health coverage (UHC) (Western Cape Department of Health [WCDoH] 2023). Disability and community-based rehabilitation (CBR) are neglected public health issues because of the dominance of vertical disease-specific primary healthcare programmes (Louw et al. 2023; The Lancet Public Health 2021). Disability is an umbrella term for impairments, activity limitations and participation restrictions arising from the interaction between a person’s health condition(s) and context (environmental and personal factors) (World Health Organization [WHO] 2012). Community-based rehabilitation is not the same as acute, transitional care and outreach rehabilitation. It is a community development strategy that creates equal opportunities for rehabilitation, poverty reduction and social inclusion for persons with disabilities and their families (WHO 2010). Community-based rehabilitation contributes to UHC and health system strengthening because it addresses the access to medical care, rehabilitation, health promotion, prevention and provision of assistive devices through intersectoral liaison at a primary level and reduces the burden of care at secondary and tertiary levels of care (WHO 2017). The delivery of CBR in COPC requires dedicated teams of rehabilitation professionals and appropriately trained rehabilitation care workers (RCWs). While outreach services might provide a continuum of care for persons with disabilities after discharge from acute secondary and tertiary levels of care or from transitional care facilities, a CBR approach would be better aligned with the fundamental principles of COPC (Philpott, McClaren & Rule 2020). In this article, we share opinions about the training of RCWs to strengthen the health system through CBR as a component of COPC.

In 2012, a provincial department of health and a local university launched an initiative aimed at developing a mid-level disability-inclusive health workforce. The aim of the initiative was to bridge the gap of suitably trained RCWs to address the public health and disability needs of communities within the primary healthcare system. The primary healthcare system is being re-engineered to align with the COPC approach. A 1-year Higher Certificate in Disability Practice (HCDP) was designed by a team of rehabilitation professionals and disability practitioners and registered at level 5 of the South African National Qualifications Framework. The first cohort of 28 students graduated in 2014. A total of 120 RCWs have graduated to date, most of whom are employed in government posts or by Non-Profit Organisations (NPOs) with their salaries paid by government.

There are five courses in the HCDP curriculum. The main learning outcome for each course is presented hereunder:

- **Inclusive Development and Agency:** To understand CBR as a strategy for inclusive development that promotes the rights of persons with disabilities and to implement strategies and actions that remove barriers and enable participation.
- **Disability Information, Management and Communication Systems:** To know basic health information systems and implement management and communication systems in relation to care pathways for persons with disabilities across sectors.
- **Promoting Health and Well-being:** To understand primary healthcare and assist in the design and implementation of disability prevention and health promotion actions.
- **Health and Functional Abilities:** To understand human development and common health conditions so that clients with disabling impairments and activity limitations can be identified and screened. To provide basic rehabilitative interventions provided that facilitate participation in the areas of living, learning, working and socialising.
- **Work Integrated Practice Learning:** To provide practice-learning opportunities that integrate knowledge and skills related to the above courses.

The aim of our opinion article is to advocate for the training of RCWs as essential and valuable human resources to COPC. Our article is based on a study that investigated the contribution of the HCDP graduates towards strengthening rehabilitation in community-based services at a primary level of care. After receiving ethical approval from the Human Research Ethics Committee (HREC) at UCT (064/2023), we conducted a collaborative inquiry with the CBR team servicing two sub-districts in a city metropole and a rural sub-district. Participants in the metropole included eight RCWs who are alumni of the first cohort of the HCDP programme; eight persons with disabilities who received their services; one social worker; three occupational therapists; and one speech and language therapist who supports and supervises the RCWs. Participants in the rural sub-district included one unemployed RCW who is also an alumnus of the HCDP, one community development worker, two persons with disabilities, two mothers of children with disabilities, one occupational therapist and one speech and language therapist. The therapists provide facility-based rehabilitation services at primary-level health clinics in the rural sub-district.

Textual and numerical data were gathered during 4-h and 6-h workshops held once a month with the participants. The discussions were recorded and transcribed.

The participants also completed and discussed a checklist of RCW services based on competencies acquired through the HCDP curriculum.

Our article presents the findings of one of the four workshops. In the workshop, the participants completed a checklist of RCW services based on competencies acquired through the HCDP curriculum. Data were analysed using deductive coding and triangulated with the WCDoH Position Statement on COPC (WCDoH 2023) and the National Framework and Strategy for Disability and Rehabilitation Services (National Department of Health 2015).

## Findings

**FIGURE 1:**
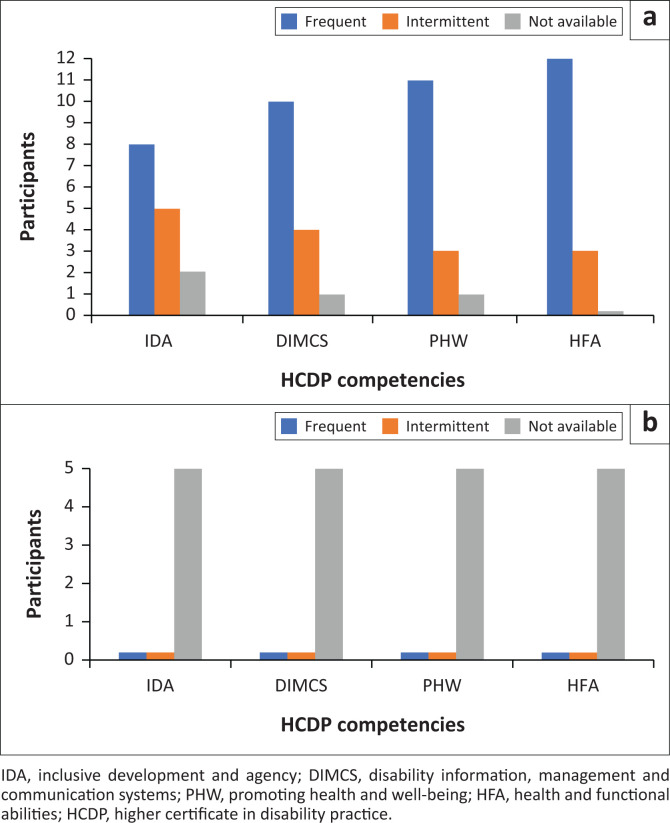
Findings of the checklist of services delivered by rehabilitation care workers linked to higher certificate in disability practice competencies: (a) Mitchells Plain and Klipfontein (*N* = 15); (b) Witzenberg (*N* = 15).

Figure 1 illustrates participants’ perspectives on services delivered by RCWs using competencies acquired through four of the HCDP courses (IDA, DIMCS, PHW and HFA [Health and Functional Abilities]). Competencies were consolidated during WIPL and subsequent practice as members of the CBR team. The blue columns in the metropole data indicate that RCWs frequently implement the knowledge and skills they acquired. The yellow columns indicate that they intermittently implement the knowledge and skills based on emergent needs and varying circumstances and populations within a sub-district. The grey column indicates that 1–2 participants felt that the services were not available. The rural data indicate that none of the RCW services are available. This is not surprising given that the one HCDP-graduated RCW is unemployed and that services by the rehabilitation professionals are facility-based at the primary care clinic level.

Table 1 presents the perspectives of participants on the perceived differences in the scope of practice of community health workers (CHWs) and home-based carers (HBCs) who do *not* have the HCDP qualification (column A) and those that *do* have the HCDP qualification (column B). These reflections are based on personal experiences of the differences, given that participants were employed as CHWs or HBCs prior to completing the HCDP. The insights are significant because current policy directives indicate that nurse-led, ward-based outreach teams of CHWs will deliver comprehensive primary care services inclusive of rehabilitation (WCDoH 2023).

**TABLE 1:** Differentiating scope of practice – According to the community-based rehabilitation.

CHW and HBC	CBR elements	Tasks performed
Without HCDP	Health – Medical care	Burden of disease Curativee
		Nurse supervision BP
		HIV and/or TB
		Glucose testing
		Wound care
		Basic limb movement and/or exercises
		Breast examination
	Health – Prevention	Medication compliance
	Health – Personal assistance	Do task FOR person – Individual focus
		Bathing
		Grooming
	Empowerment – Advocacy and communication	Health campaigns
With HCDP	Health – Medical care	Burden of disability
		Developmental (goal driven, limited)
	Health – Prevention	Do task WITH person – Advocacy
		Holistic care: individual & family focus
		Hearing screening Mental health support
		H – rehab
	Health – Promotion	Basic counselling
		Play activities
		Wellness promotion
		Referral pathways
	Health – Rehabilitation	Independence and/or functioning in ADL
		Rehabilitation screening
	Health – Assistive devices	Assistive devices (incl. hearing aids): Use and repair
	Social – Recreation and sports	Sport and recreation opportunities
	Social – Relationships	Basic counselling
	Livelihood – Skills development	Livelihood opportunities
	Empowerment – Advocacy and communication	Disability advocacy: Inclusion and participation
	Empowerment – Community mobilisation	Asset mapping: Intersectoral links

HCDP, higher certificate in disability practice; HIV, human immunodeficiency virus; TB, tuberculosis; BP, blood pressure; CHW, community health worker; HBC, home-based carers; ADL, activities of daily living; CBR, community-based rehabilitation.

The findings in Figure 1 and Table 1 provide evidence that the HCDP extends the scope of practice of CHWs and HBCs and contributes to the strengthening of rehabilitation services at the primary level of care.

### Theme 1: Enhancing functional abilities

Our findings highlight that the content and practical rehabilitation skills or techniques taught in the Health and Functional Abilities course equip the RCW to provide a wide range of rehabilitation activities and facilitate function and participation. Furthermore, it is evident that RCWs also provide holistic care, which includes mental health and well-being:

‘RCW comes every week, help me with the arm, stretching exercise. Service is excellent, they do everything on my body, all round exercise, leg arm body turning. Train me on washing, do other activities, lifting weights. The team is helpful, they work so much. RCWs motivate me to do something with my life, provide basic counselling, inspire me. They encourage me to drink the mental health medication.’ (Middle aged, female, person with a disability)

‘RCWs zoom into specific things that will inform therapist that – involves participation, engagement, inclusion and functional things.’ (Female, non-disabled person, rehabilitation care worker)

### Theme 2: Promoting health and well-being

Our results show that all the courses are integrated into practice, for example, the evidence hereunder indicates that RCWs apply the content taught in the PHW course and use a holistic approach to address factors related to health and social well-being:

‘[*RCWs focus on*] prevention and promotion, holistic approach in addition to health. Address everything challenging the patient, anything contributing to the client, do school, livelihood, medical, social, empowerment etc.’ (Female therapist in a rural area)

‘RCWs can plan, implement when given a project, can run a group. They are trained to think of group dynamics, make activities specific to accommodate attendees’ needs.’ (Female, non-disabled person, rehabilitation care worker)

### Theme 3: Strengthening continuity of care

Evidence suggests that because the CHWs in the rural areas are not aware of the HCDP programme, their knowledge and skills are limited with regard to rehabilitation and disability. They are not necessarily able to identify when a client requires a referral to facilitate their continuity of care. The continuity of care is emphasised strongly within all courses in the HCDP programme:

‘They [*CHWs*] are not trained. Like for example, I know that they sometimes look at the weight and the growth of the baby, those are the things that they can identify, but with regards to rehab, they can only identify whether a person is in the wheelchair, or the person is using a crutch but all the other things they are not able to identify and refer to us. I definitely think [*HCDP*] training for them [*CHWs*] would be very needed … she was telling us about the programme, I was so surprised I never heard of it. It sounds amazing.’ (Female, non-disabled person, therapist)

## Discussion

The three themes show a clear alignment between the competences of the RCWs and their practice, which contributes to strengthening the implementation of the COPC framework. Our findings show that the metropole is better resourced than the rural areas; thus, the need exists for having RCWs as part of the rehabilitation team in rural areas. This may be attributed to the fact that the programme development and initial training were initiated and funded by the WCDoH. However, there are a limited number of RCW posts and trained RCWs available to deliver rehabilitation services to persons with disabilities and their families at a primary level. We propose that CHW skills be upgraded through teaching and learning pedagogical innovations such as the HCDP and that vacant nurse assistant and/or general assistant posts be allocated to posts for RCWs. We are of the opinion that there are limited career development opportunities for RCWs and suggest articulation into a 2-year diploma programme that enables RCW services with wider intersectoral collaboration in line with the education and livelihood components in the CBR guidelines (WHO 2010).

## Conclusion

The HCDP programme is a critical human resource development strategy that ensures that persons with disabilities and their families across the life course receive basic rehabilitation and community support systems so that they can live productive, healthy and meaningful lives. Evidence suggests that upskilling CHWs with HCDP training strengthens community-orientated primary care services within the metropole, and the same approach of prioritising training of CHWs in the rural area will be helpful in improving rehabilitation services in rural areas.
